# Ontogenetic niche shifts in a locally endangered tree species (*Olea europaea* subsp. *cuspidata*) in a disturbed forest in Northern Ethiopia: Implications for conservation

**DOI:** 10.1371/journal.pone.0256843

**Published:** 2021-09-30

**Authors:** Mekdes Ourge Wegasie, Kari Klanderud, Ørjan Totland, Katrine Eldegard

**Affiliations:** 1 Faculty of Environmental Sciences and Natural Resource Management, Norwegian University of Life Sciences, Ås, Norway; 2 College of Dryland Agriculture and Natural Resources, Mekelle University, Mekelle, Ethiopia; 3 Department of Biology, University of Bergen, Bergen, Norway; Qingdao Agricultural University, CHINA

## Abstract

Understanding the responses of different ontogenetic stages to environmental and human disturbance factors is essential for developing efficient conservation strategies for endangered plant species. We examined how three ontogenetic stages of a locally endangered tree species, *Olea europaea* subsp. *cuspidata*, responded to environmental factors and human disturbance in Hugumburda dry Afromontane forest in Ethiopia. We counted individual seedlings, saplings and adults of *O*. *europaea* in 70 20 × 20 m quadrats over ca. 2.8 ha, and measured biotic (woody species richness, canopy cover, aboveground tree biomass, herbaceous cover), abiotic (soil and topographic variables), and human disturbance factors (logging and tracks). To detect ontogenetic niche shifts, we compared observed vs. simulated locations of trees in the three life stages and how they related to the environmental and human disturbance factors. We found that the population structure of *O*. *europaea* showed generally low recruitment, with few seedlings per hectare compared with the abundance of saplings and adults. The probability of finding *O*. *europaea* individuals was influenced by biotic (woody species richness) and abiotic (soil depth, slope) environmental conditions and human disturbance (logging intensity), but the direction, strength and shape of the relationships differed between seedling, sapling and adult life stages, indicating ontogenetic niche shifts. All life stages showed a positive relationship with elevation. The observed environmental niches of the different lifestages of *O*. *europaea*, and their association with human disturbance levels, should be considered when conservation strategies are developed for this species. Human disturbance in terms of logging decreases the abundance of saplings, but may facilitate emerging seedlings through creation of gaps with improved light conditions. Recruitment is, however, very low in the study area, and seedlings should be protected from browsing to enhance survival. Woody species richness in general should be conserved to optimize conditions also for *O*. *europaea* saplings.

## Introduction

Niche requirements of plants may change through their different life stages [1, 2]. This can be due to differences in micro-site suitability, for instance, the requirements of seedlings (i.e. the regeneration niche) can differ from that of later life stages, even within the same population [3, 4]. Such ontogenetic shifts start with niche differentiation taking place early in a plant’s life [5]. Different life stages may also respond differently to interactions with neighbour vegetation, as early life stages may be more prone to biotic interactions, such as competition, than later life stages [6]. Regeneration of the plant population depends on the availability of viable seeds, and how the germination success and survival of seedlings are related to local environmental factors, such as light reaching the ground, soil moisture and nutrient content, and human and animal disturbance [3, 6, 7–9]. In contrast, according to the unified neutral theory [10], niche shifts in plants are less relevant, because plants, unlike animals, commonly share similar resources from the environment [11]. Thus, the concept of ontogenetic niche shifts has been less studied in plant ecology [but see 1, 2, 8, 12]. In contrast, niche shifts between consecutive stages is common and well-studied in animals [13, 14]. The reasons are complex but perhaps reflect the difference that unlike many animals, which are mobile and can move to environments that suit their needs at different developmental stages, plants are sessile and must adjust at each stage to the environment in which they find themselves. Ontogenetic niche shifts in plants therefore reflect the intersection of changes in environments over time; developmental changes in the plants as they mature, and whether these expand or contract a species’ niche space; and any resulting differentials among individuals in growth rates and survival. Also, the effect an individual plant may have on the environment may change over time [15].

Examining the responses of different ontogenetic stages to abiotic and biotic environmental and disturbance factors may give insights into how niche shifts can occur between different life stages of the same plant species [16, 17]. For instance, in an ontogenetic niche shift experiment on three *Vaccinium* shrub species in a boreal forest, the niche requirements of juvenile *Vaccinium vitis-idaea* were different from those of adults [18]. Studies that include sequential life stages are therefore important for understanding plant population dynamics and applying this knowledge to conservation measures [8]. In addition, environmental factors play a significant role in ontogenetic niche shifts and are further important in understanding plant distribution and abundance [19, 20]. For instance, in a study of 12 montane-forest tree species in Slovakia, the abundance of seedlings varied significantly from those of saplings and adults in response to elevation, temperature, and precipitation [21]. Environmental factors can also limit niche shifts in exotic plant invasions and distribution [22]. Thus, to be able to both protect endangered species and to decrease the abundance of invasive species, understanding how ontogenetic shifts are related to environmental factors and human disturbance is vital for developing effective conservation strategies [8].

Most previous studies of ontogenetic niche shifts in plants have used analysis of variance or generalized linear (mixed) model approaches [8, 12, 17, 18, 23]. In contrast, canonical correspondence analysis have been used to analyze whether various ontogenetic stages differed in their response to environmental conditions [9, 24]. Comparing observed abundances among life stages is not straightforward because the sizes of individuals differ substantially as they mature. To overcome this problem, we applied a novel analytic approach, using both empirical and simulated observations (random relocations of the observed data) as inputs.

In this paper, we report the population structure and ontogenetic shifts of a locally endangered tree species, *Olea europaea* subsp. *cuspidata* (hereafter *O*. *europaea*). This species is particularly interesting regarding ontogenetic niche shifts because of its ability to grow in both dry and moist environments [25]. It is locally endangered because of overexploitation for wood for multiple purposes [26–28], and there is a need for more research to understand it’s population status, and to understand how it can be best conserved [29]. We asked the following questions: (i) What is the current population structure of *O*. *europaea* in the studied forest? (ii) How are the various life stages of *O*. *europaea* related to selected biotic and abiotic environmental and human disturbance factors? (iii) Can the distribution of adult *O*. *europea* be predicted by the same environmental and human disturbance variables as those for saplings and seedlings? As *O*. *europaea* is commonly harvested for wood, we predict that the number of reproductive adult trees, and thus seeds emerging to seedlings, can be the bottleneck for this locally endangered species. Seedlings and saplings are likely more dependent on abiotic conditions providing good conditions for seedling emergence and establishment, such as soil moisture and soil depth, and more vulnerable to biotic interactions, such as competition for light from the vegetation canopy, and animal disturbance. Adults, on the other hand, are likely more prone to logging, and therefore expected to be more likely to occur in less accessible areas and steeper slopes [30]. Thus, different environmental and disturbance variables are likely needed to predict the distribution of adults vs. younger life stages of *O*. *europaea*.

To test these hypotheses, we performed and extensive field sampling of three life stages of *O*. *europaea* in 70 plots differing in environmental and disturbance factors in the dry Afromontane Hugumburda forest in Northern Ethiopia.

## Materials and methods

### Study species and area

In Ethiopia, *Olea europaea* subsp. *cuspidata* occurs mostly in dryland areas, and it is a characteristic species of dry Afromontane forests, with an elevational range of 1250 to 3100 m a.s.l. The height of adult trees rarely reaches 25 m [31, 32]. Once established, the tree grows well in both warm and cold climate conditions [33]. *Olea europaea* wood is commonly used for fencing, fuelwood and making charcoal, and for manufacturing farm implements and home furniture. Because of its multipurpose use, the species has become locally endangered in Ethiopia [25, 27, 28, 34, 35].

The study area is in Hugumburda dry Afromontane forest (12° 22’–12° 42’ N, 39° 28’–39° 40’ E), which is one of the National Forest Priority Areas, located in the Tigray Province in the northern part of Ethiopia (S1 Fig). The topography is rugged and mountainous, with elevation ranges from 1796–2772 m a. s. l. The soil depth is shallow, and mostly consists of leptosoil and regosol soils [36]. Mean annual temperature is 15.4°C with a daily temperature range from 14.6 to 22.4°C. With a mean minimum temperature of 10.7°C, October is the coldest month. June is the hottest, with a mean maximum of 34.3°C [37]. Mean annual rainfall is 705–986 mm [38]. Hugumburda is a secondary forest [36] and includes some residential areas and agricultural fields along the borders. Previous land use (1950s) was commercial timber production, but the forest has now been protected since 1993. There is wood collection and livestock (cattle and goats) grazing in the area [27].

Our study area covered ca. 2.8 ha of the forest, comprising high-elevation natural forest and plantations. The structure of the forest mainly comprises trees, bushes, lianas, and herbs. Dominant woody species are *Juniperus procera*, *O*. *europaea* subsp. *cuspidata*, *Nuxia congesta*, *Rhus glutinosa*, *R*. *natalensis*, and *Olinia rochetiana* [37].

### Field data collection

In 2015, seventy 20 × 20 m plots were distributed along twelve elevational transects perpendicular to three local terrain ridges covering forested slopes from 2233 to 2503 m a.s.l. (S1 Fig). The distance (geographical) between neighboring transects was ca. 150 m and between plots ca. 100 m. In each 20 × 20 m main plot, we measured stem diameter at breast height (DBH) and the heights of all adult *O*. *europaea* individuals [39]. We also counted and measured the diameter of all *O*. *europaea* stumps as a measure of logging activity. The abundance, height and diameter of *O*. *europaea* saplings were recorded in 5 × 5 m sub-plots, placed in the center of each main plot. The number and heights of *O*. *europaea* seedlings were recorded in five small 1 × 1 m plots, one placed in the center of each main plot and four at the corners of the 5 × 5 m sub-plots. To classify life stages of *O*. *europaea* into adult, sapling or seedling we used the following criteria: adults (DBH > 2 cm, height > 1.3); saplings (DBH < 2 cm, height > 0.3 ≤ 1.3 m) and seedlings (DBH < 2 cm, height ≤ 0.3 m). See [27, 30] for more details on the sampling design).

We sampled biotic, abiotic and human-disturbance variables in the same plots as the *O*. *europaea* data. For biotic factors, we recorded woody species richness by counting the number of all woody shrub, tree and climber species other than *O*. *europaea* (in total 39 species, see S1 Table). Local botanists assisted in identifying the species in the field. We recorded the height and DBH of all the woody species, from which we calculated aboveground biomass (hereafter biomass). We estimated tree canopy cover by eye as percentage cover to the nearest 1%. We also visually estimated the percentage cover of herbaceous species in each 1 × 1 m plot.

Slope and elevation were measured in the center of each main plot with a clinometer and a handheld GPS (Garmin eTrex Euro with accuracy 5–10 m), respectively. For each of the 70 main plots, a soil core sampler was used to collect two separate, 0–15 cm depth, soil samples: one for measuring soil moisture content; the other for chemical analysis. For the soil chemical analyses, we used a composite of five samples collected at the center of each 1 × 1 m plot. The soil samples were sealed in a double plastic bag to preserve the moisture. All soil samples were transported to Mekelle Agricultural Research Center laboratory in Mekelle. Soil moisture was measured gravimetrically [40] (Gardner, 1965). The composite soil samples were air-dried at room temperature and sieved through a 2 mm mesh. Soil pH (a 1:2.5 soil:H_2_O mixture) and soil organic matter (Walkley-Black method) were measured using standard techniques [41]. Soil depth was measured in the field by digging a pit at the center of the main plot down to bedrock [see also 30].

To quantify human disturbance in each main plot, we measured the total length (m) of tracks with a measuring tape, and estimated logging intensity by counting the number of cut *O*. *europaea* stumps (hereafter logging). We used these variables as indicators of human disturbance. Permission to perform field sampling was given by the Forest Managing Authority of Hugumburda forest.

### Data analysis

To assess *O*. *europaea* population structure, we sorted all the individuals into 14 diameter and 12 height classes. The diameter classes increased by 1.2 cm increments between classes from class 1 (< 0.8 cm) to 14 (> 16.3 cm). The height classes increased by 0.9 m increments between classes from class 1 (< 0.3 m) to 12 (> 10.3 m). The number of individuals in each class was calculated and the diameter and height distributions presented as frequency histograms.

We calculated the above-ground biomass of all adult tree species other than *O*. *europaea* recorded in the main plots by applying DBH, height (H) and wood density (WD) to the following allometric equation developed for tropical trees [42]:

$$\text{Above}\;\text{ground}\;\text{biomass}=0.0673*{\left(\text{WD}*{\text{DBH}}^{2}*\text{H}\right)}^{0.976}$$

Average wood density (0.612 g/cm^3^) for all tree species was obtained from the global wood density database [43].

We used Non-metric Multidimensional Scaling (NMDS) to investigate the associations between life stage (seedlings, saplings and adults), and the environmental and disturbance factors. All environmental variables and the abundance of individuals in each life stage were standardized to make them comparable. The Bray Curtis dissimilarity index was used to define an appropriate number of dimensions (k) in the NMDS analyses. We performed ordinations of progressively higher dimensions (k = 2–4), which produced stress values of 0.215, 0.151 and 0.110, respectively. The NMDS analyses were performed through the metaMDS function in the vegan package in R [44], with 1000 random starting points. The importance of each environmental variable was assessed from the squared correlation coefficient (R^2^) between the environmental variable and the ordination axes. Statistical significance (p-value) of both vector and factor variables was based on random permutations of the data. To assess which life stages of *O*. *europaea* abundance were associated with specific environmental factors (biotic, abiotic and human disturbance), we used the envfit command to place the vectors (i.e. the abundance of life stages) of environmental gradients onto the NMDS ordination. To reduce complexity, we limited our data presentation to three dimensions (linear fit: R^2^ = 0.84).

NMDS, like other ordination techniques, indicates differences among seedlings, saplings and adults in their relationships with the various environmental factors, but it does not provide a rigorous statistical test of the strength of these associations. Therefore, we compared our empirical data with a simulated dataset consisting of random relocations of the sampled seedling, saplings and adult individuals from the 70 plots.

To examine if the abundance of seedlings, saplings and adults were influenced by the same environmental and disturbance variables, we fitted a generalized linear model (GLM) with a binary response in which Y = 0 for simulated observations, and Y = 1 for empirical observations. We assumed a binomial distribution of errors and used a logit link function (logistic regression). The model estimated the likelihood of an empirical observation responding to the explanatory variables. Hereafter, we refer to the response variable as the probability of finding *O*. *europaea*. To reduce model complexity, we pre-selected variables by fitting single variable models of the relationship between the response variable and each of the candidate explanatory variables, for each ontogenetic stage. Candidate explanatory variables included both biotic (woody species richness, total aboveground biomass of all tree species except *O*. *europaea*, canopy cover and cover of herbaceous plants), abiotic (soil depth, soil pH, soil organic matter, soil moisture, slope, elevation) and human disturbance variables (tracks and logging). We fitted a full model with those explanatory variables that were significantly related (p < 0.05) to the response variable for at least one of the ontogenetic stages, using the two-way interactions between each of these variables and ontogenetic stage as explanatory terms. A significant interaction (p < 0.05) between an ontogenetic stage and an environmental variable was interpreted as indicating a difference among the ontogenetic stages in their response to the environmental variable, i.e., an ontogenetic niche shift.

We carried out model selection by an information criterion based approach, selecting the model with the smallest Akaike’s information criterion (AIC) value as our final model. The general variance inflation factor (GVIF) values were < 5 for all combinations of the explanatory variables in the final models. The model was fitted using the MASS package in R 3.2.5 software [45].

## Results

**Fig 1:**
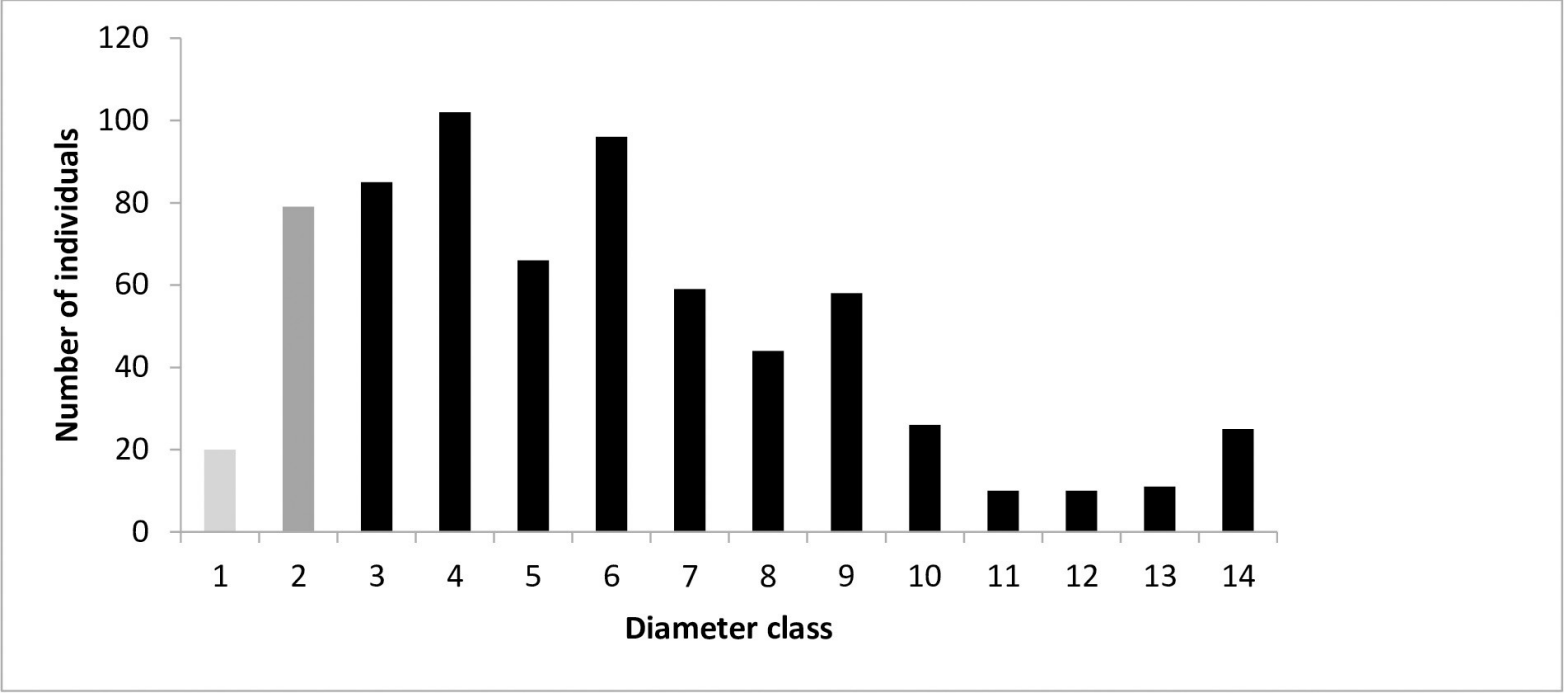
Diameter class distribution of *O*. *europaea* subsp. *cuspidata* in Hugumburda dry Afromontane forest in northern Ethiopia (1 = < 0.8; 2 = 0.8–2.0; 3 = 2.1–3.3; 4 = 3.4–4.6; 5 = 4.7–5.9; 6 = 6–7.2; 7 = 7.3–8.5; 8 = 8.6–9.8; 9 = 9.9–11.1; 10 = 11.2–12.4; 11 = 12.5–13.7; 12 = 13.8–15.0; 13 = 15.1–16.3 and 14 >16.3 cm). Seedlings, light grey bar; saplings, dark grey bar; and adult trees, black bars.

**Fig 2:**
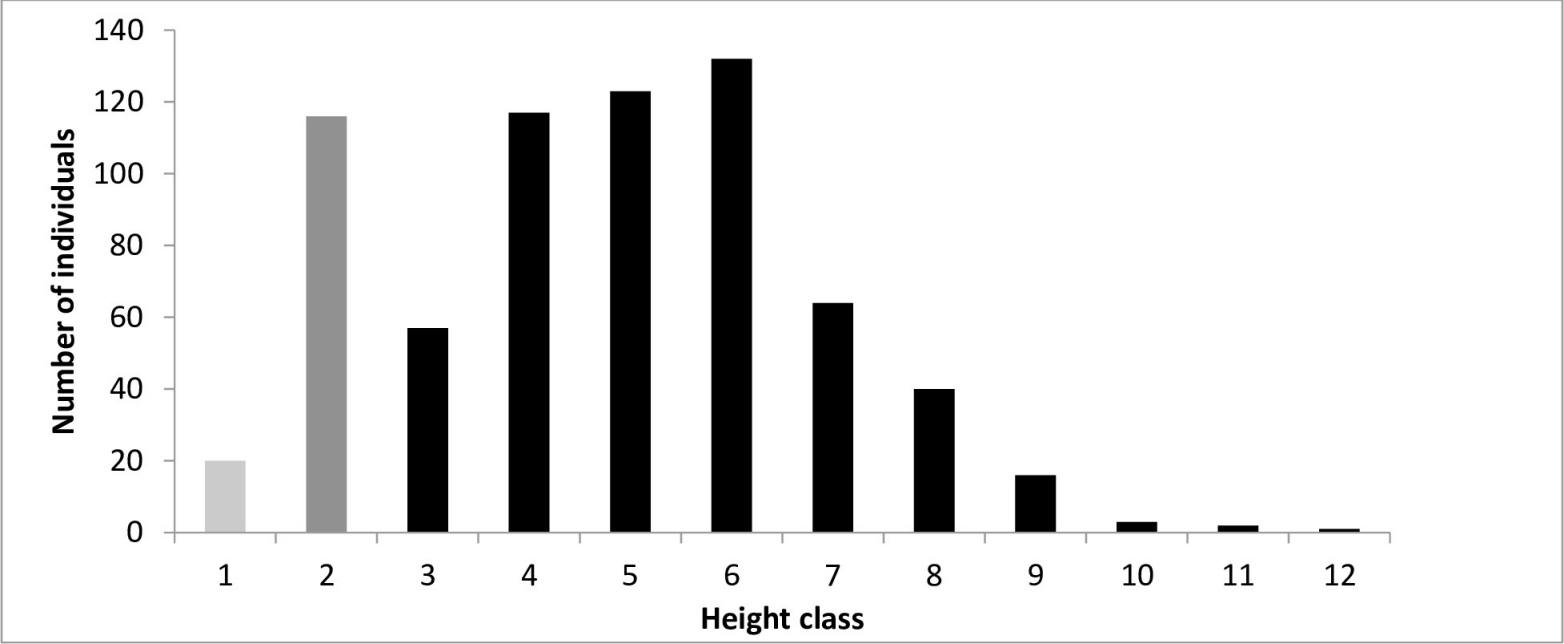
Height class distribution of *O*. *europaea* subsp. *cuspidata* in Hugumburda dry Afromontane forest in northern Ethiopia (1 < 0.3; 2 = 0.3–1.3; 3 = 1.4–2.3; 4 = 2.4–3.3; 5 = 3.4–4.3; 6 = 4.4–5.3; 7 = 5.4–6.3; 8 = 6.4–7.3; 9 = 7.4–8.3; 10 = 8.4–9.3; 11 = 9.4–10.3; 12 = > 10.3 m). Seedlings, light grey bar; saplings, dark grey bar; and adult trees, black bars.

The abundance of *O*. *europaea* seedlings (7 ± 5 individuals ha^-1^) was much less than either the sapling (79 ± 25 individuals ha^-1^) or adult stages (219 ± 39 individuals ha^-1^). The diameter distribution of the population also indicated that the number of seedlings was low compared with the older sapling and adult stages. Most individuals were found in diameter classes 2–9 (Fig 1) and in height classes 2–7 (Fig 2). There was a sharp decline in number of individuals higher than 5.3 m (height classes 7 and above, Fig 2).

**Fig 3:**
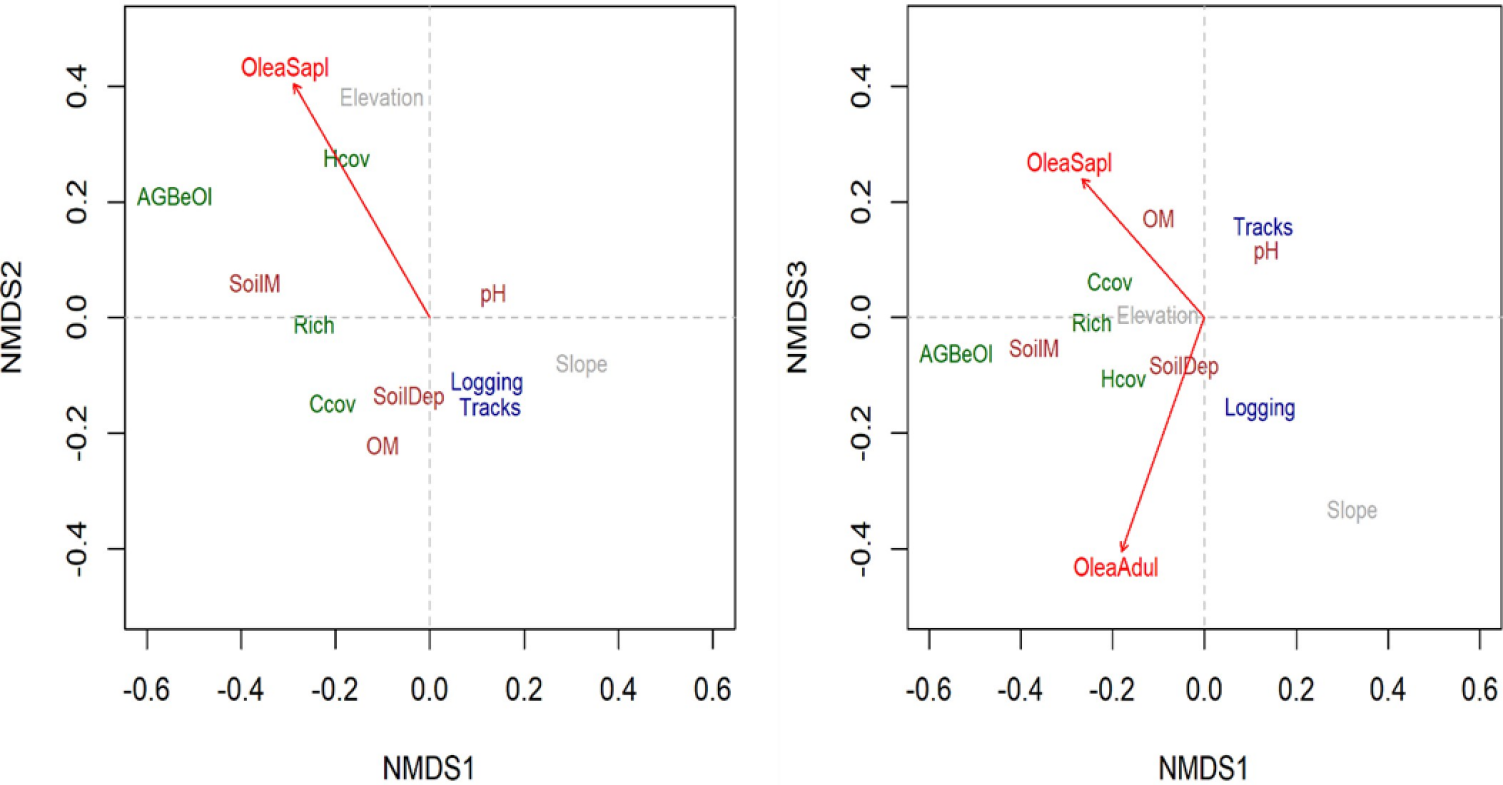
Nonmetric multidimensional scaling (NMDS) ordination of *O*. *europaea* life stages versus environmental conditions and human disturbance factors in Hugumburda dry Afromontane forest in northern Ethiopia. *O*. *europaea*. Life stages are shown in red, topographic factors are shown in dark grey, soil factors in brown, biotic factors in dark green and disturbance factors in dark blue. *O*. *europaea* adult (OleaAdul), *O*. *europaea* sapling (OleaSapl), organic matter (OM), canopy cover (Ccov), richness (Rich), track length (Tracks), elevation (Elevation), slope (Slope), number of *O*. *europaea* stumps (Logging), herbaceous cover (Hcov), aboveground biomass of all trees except *O*. *europaea* (AGBeOl), soil moisture (SoilM), soil depth (SoilDep) and human footpaths (Tracks).

The NMDS ordination plots show how the seedling, sapling and adult stages of *O*. *europaea* are associated with the environmental and disturbance factors. *Olea europaea* seedlings were not significantly associated with any of the NMDS axes. In contrast, saplings and adults were, but in different ways. Saplings were evidently related to the second and third NMDS axes (positively associated with elevation and herbaceous cover on NMDS 2, and with soil organic matter on NMDS 3, and negatively associated with logging, slope and tracks on both axes (Table 1 and Fig 3). Conversely, adults seemed to be positively associated with slope, slightly less positively linked with herbaceous cover and logging, and negatively associated with tracks and pH, all on NMDS 3 (Table 1 and Fig 3).

**Table 1:** Squared correlation coefficients and associated p-values from permutation tests of the associations between *O*. *europaea* life-stage abundances and NMDS axes.

Environmental Variables	NMDS 1 vs NMDS 2		NMDS 1 vs NMDS 3	
	r^2^	p	r^2^	p
Seedlings	0.0152	0.659	0.0122	0.694
Saplings	0.1997	**0.005**	0.1180	**0.023**
Adult	0.0557	0.205	0.1778	**0.007**

Figures in bold are statistically significant at p < 0.05.

**Fig 4:**
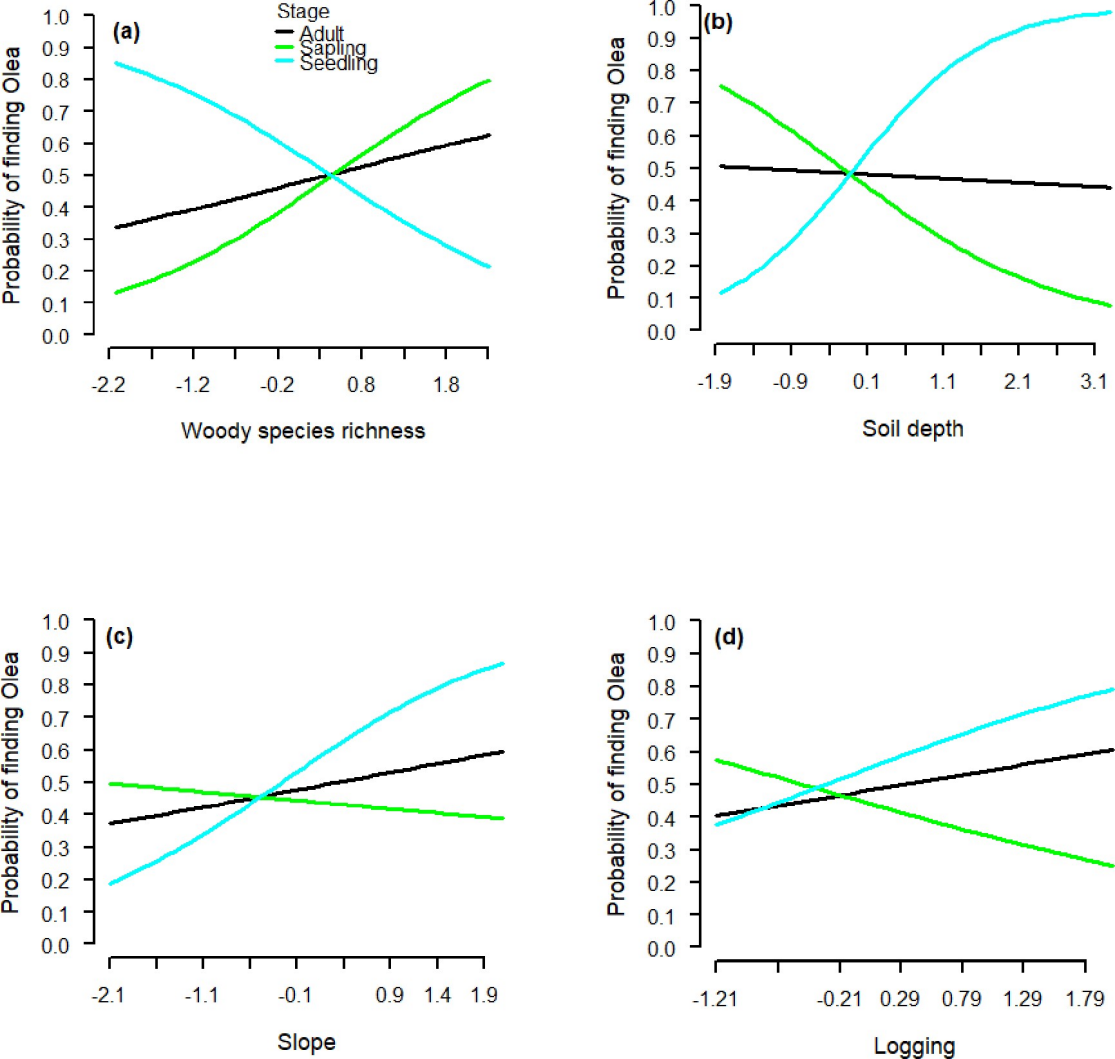
Estimated relationships between the probability of finding *O*. *europaea* and woody species richness, soil depth, slope and logging, for different ontogenetic stages (adult, sapling, seedling; see Table 2) in Hugumburda dry Afromontane forest in northern Ethiopia. Note that the explanatory variables were standardized before analysis and plotting. Observed values ranged from 6 to 24 species for woody species richness, from 0.01 to 1.20 meters for soil depth, from 0.03 to 0.94 degrees for slopes, and logging intensity from 0 to 13 for number of stumps per 20 × 20 m observation plots for logging.

The probability of finding *O*. *europaea* in at least one of its ontogenetic stages was significantly associated (p < 0.05) with the environmental factors woody species richness, soil depth, soil moisture, elevation, slope, and the human disturbance factor logging (Table 2). The significant interactions ontogenetic stage and environmental variables, revealed by the GLMs, indicated ontogenetic niche shifts (Table 2). Woody species richness was positively related to adults and saplings, but negatively related to seedlings (Fig 4A). Opposite trends were found for soil depth, which was positively related to seedlings and negatively related to saplings, and had no clear impact on adults (Fig 4B). Both slope and logging intensity were also positively related to seedlings and negatively related to saplings, and also positively related to adults (Fig 4C and 4D).

**Table 2:** Results of a logistic regression model, estimating how the probability of finding *O*. *europaea* depends on ontogenetic stage (adults [reference level], sapling, seedling) and environmental conditions and human disturbance (logging intensity) in Hugumburda dry Afromontane forest in northern Ethiopia.

	Estimate	SE	z	p
Intercept (Adult)	-0.136	0.07	-2.1	**0.037**
Sapling	-0.139	0.12	-1.0	0.317
Seedling	0.275	0.45	0.6	0.542
Woody species richness	0.269	0.08	3.2	**0.001**
Soil depth	-0.051	0.09	-0.6	0.554
Elevation	0.213	0.07	3.1	**0.002**
Slope	0.217	0.07	3.2	**0.001**
Logging intensity	0.253	0.08	3.3	**0.001**
Seedling × Woody species richness	-0.958	0.61	-1.6	0.114
Sapling × Woody species richness	0.467	0.17	2.8	**0.006**
Seedling × Soil depth	1.207	0.57	2.1	**0.035**
Sapling × Soil depth	-0.646	0.17	-3.8	<**0.001**
Seedling × Slope	0.580	0.45	1.3	0.201
Sapling × Slope	-0.324	0.13	-2.4	**0.014**
Seedling × Logging intensity	0.321	0.56	0.6	0.566
Sapling × Logging intensity	-0.687	0.19	-3.6	**<0.001**

The table shows parameter estimates and associated standard errors for a binary generalized linear model, where the response had two possible outcomes Y (empirical observation) = 1 and Y (simulated observation) = 0. Simulated observations were generated by random relocations of the empirical observations of O. europaea in 70 sample plots. Figures in bold are statistically significant at P < 0.05.

Overall, the interpretation of the NMDS ordination outputs and the results from the GLMs appeared to show similar trends with respect to the influence of the environmental variables slope, logging, woody species richness and soil depth on the probability of finding saplings and adults (Tables 1 and 2 and Figs 3 and 4).

## Discussion

This study reveals ontogenetic niche shifts in *Olea europaea*. The probabilities of finding the various life-stages related differently to the suites of biotic and abiotic environmental factors that we studied. This suggests that each ontogenetic stage has a different tolerance to various ecological factors, suggesting niche differentiations in *O*. *europaea*, as has been found in another threatened tree species, *Acer opalus* subsp. *granatense* [8]. We also show that seedlings, saplings and adults of *O*. *europaea* are subject to different human pressures in the study area.

The higher probability of finding *O*. *europaea* seedlings with increasing soil depth is in line with our hypothesis, and suggests that deeper soil is important for seedlings to establish, likely because deeper soils retain more moisture. Soil depth has been shown to be an important factor for the establishment of seedlings, as for example in recently abandoned pastures and secondary forest in Costa Rica [46]. Moreover, in a seed dispersal and establishment experiment on *O*. *europaea* in Spain, soil moisture was the primary determinant of seedling establishment among several abiotic factors studied [47]. Also another study on regeneration in a dry Afromontane forest [48], recorded a higher recruitment rate of *O*. *europaea* seedlings during the wet season than in the long dry season, emphasising the importance of soil moisture.

In contrast to our hypothesis, we observed that the probability of finding *O*. *europaea* saplings decreased as soil depth increased. Because harvesting takes place predominantly at lower elevations, the abundance of both saplings and adults is greater at higher elevations, where the soil is also shallower [30]. The higher probability of finding *O*. *europaea* adults with increasing slope also suggest that it is more difficult to harvest *Olea* on steeper slopes [30]. Thus, the current abundance of saplings and adults in this forest may be determined more by human disturbance than their preferred ecological niches. This is also in line with the positive relationship between the probability of finding *Olea* adults and logging, suggesting that logging occurs primarily in forest areas were *O*. *europaea* is abundant [30]. The sharp decline in the larger diameter classes can be due to selective logging of big trees in the forest, partly supporting our hypothesis that the number of reproductive adult trees may be low. A similar trend in other montane tree species was observed in Harenna forest, southeastern Ethiopia [49].

However, the probability of finding sapling and adult *O*. *europaea* also increased with increasing woody species richness. One reason can be that a high diversity of other woody plants may create more favourable environmental conditions, thereby enhancing the survival of saplings that can grow and establish as adults [30, 50]. Such facilitation by woody species has previously been found in dry Afromontane forest, where the establishment and survival of *O*. *europaea* seedlings has been enhanced under shrubs serving as nurse plants [51, 52], and for long-time survival of the endangered tree *Acer opalus* subsp. *Granatense* in the Iberian Peninsula [53]. In our study, however, the probability of finding *O*. *europaea* seedlings at sites with higher woody species richness was substantially less than that of saplings and adults. This is partly in line with our hypothesis, as seedlings may be more vulnerable than saplings and adults to competition for light from established woody plants. This was supported by the increase in seedling abundance with logging, suggesting that logging may create gaps in the canopy letting through light for the seedlings to emerge [54].

The present population structure of *O*. *europaea* in Hugumburda reveals a much lower density of seedlings than of saplings and adults, partly in contrast to our predictions. This points to low regeneration of *O*. *europaea* compared with other Afromontane forests in the region. For instance, *O*. *europaea* seedling density in protected parts of the Dessea forest was 3113 ± 19 individuals ha^-1^ [55], which is much greater than our observations in Hugumburda (7 ± 5 individuals ha^-1^). The reason for this low regeneration could be lack of reproductive adults due to logging. Another explanation can be browsing of seedlings by livestock in the Hugumburda forest. Goats (*Capra hircus*) are the main browsers of *O*. *europaea* seedlings in the dry forests in Ethiopia [51], and we often observed them browsing in the forest during our fieldwork (Personal observations).

## Conclusions

Our approach of testing for ontogenetic niche shifts by comparing empirical data with simulated data can advance the study of ontogenetic niche shift in plant populations. Our findings suggest that there are ontogenetic niche shifts in *O*. *europaea* in the Hugumburda forest, because seedlings, saplings and adults are differently related to the measured environmental variables. However, in addition to ecological conditions, disturbance, such as livestock browsing and logging, appear to be important determinants of seedling and adult abundances. Thus, special attention should be given to protect seedlings from browsing, and at the same time limit logging of reproductive adult trees, to secure regeneration in the effort to conserve the declining population of *O*. *europaea* in Hugumburda.

## Supporting information

S1 Fig Map of study area. Location of the study area in northern Ethiopia, and locations of the 70 study plots sampled in 2015 at different elevations in the Hugumburda dry Afromontane forest, Northern Ethiopia.
(DOCX)

S1 Table Species list. List of woody species recorded as shrubs, trees or climbers in the study area in Hugumburda dry Afromontane forest in northern Ethiopia in 2015.
(DOCX)
