# Supplementary material for: Ontogenetic niche shifts in a locally endangered tree species (Olea europaea subsp. cuspidata) in a disturbed forest in Northern Ethiopia: Implications for conservation
Source: PLoS One. 2021 Sep 30;16(9):e0256843. doi: 10.1371/journal.pone.0256843 (PMC8483397; doi:10.1371/journal.pone.0256843)

**Supporting information Figure 1.** Location of the study area in northern Ethiopia, and locations of the 70 study plots sampled in 2015 at different elevations in the Hugumburda dry Afromontane forest, Northern Ethiopia


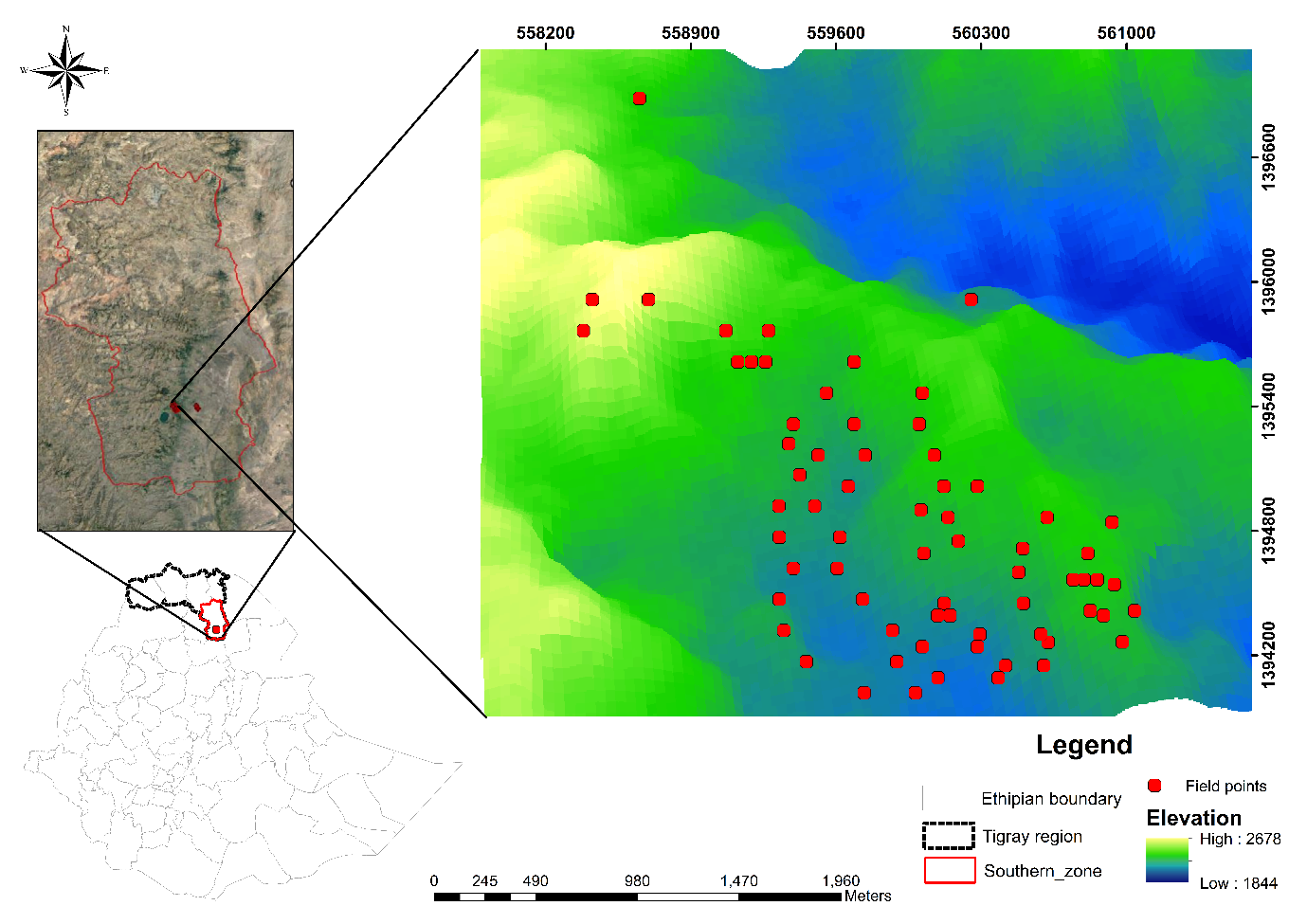

Supplement: S1 Fig — Location of the study area in northern Ethiopia, and locations of the 70 study plots sampled in 2015 at different elevations in the Hugumburda dry Afromontane forest, Northern Ethiopia. (DOCX) [file pone.0256843.s001.docx]
