# Supplementary material for: Ontogenetic niche shifts in a locally endangered tree species (Olea europaea subsp. cuspidata) in a disturbed forest in Northern Ethiopia: Implications for conservation
Source: PLoS One. 2021 Sep 30;16(9):e0256843. doi: 10.1371/journal.pone.0256843 (PMC8483397; doi:10.1371/journal.pone.0256843)
Supplement: S1 Table — List of woody species recorded as shrubs, trees or climbers in the study area in Hugumburda dry Afromontane forest in northern Ethiopia in 2015. (DOCX) [file pone.0256843.s002.docx]

| **Supporting information S1Table**  **S1 Table** List of woody species recorded as shrubs, trees or climbers in the study area in  Hugumburda dry Afromontanbe forest in northern Ethiopia in 2015. | | |  |
| --- | --- | --- | --- |
|  |  |  |  |
| **Family** | **Species** | **Vernacular (Tigregna)** | **Growth form** |
| **Shrubs** |  |  |  |
| Oleaceae | *Jasminum grandiflorum* (R.Br.ex.Fresen.) P.S.Green | Tselim-habi | shrub |
| Apocynaceae | *Carissa edulis* (Forssk.) Vahl | agam | shrub |
| Solanaceae | *Solanum schimperianum* Hochst. ex A.Rich. | Berbereawald | shrub |
| Lamiaceae | *Otostegia fruticosa* (Forssk.) Schweif.ex Penzing | chamo | shrub |
| Fabaceae | *Calpurnia aurea* (Ait) Benth. | hatsawts | shrub |
| Euphorbiaceae | *Clutia abyssinica* Jaub. & Spach. | hirtimtimo | shrub |
| Myricinaceae | *Myrsine africana* L. | kachemo | shrub |
| Fabaceae | *Pterollobium stellatum* (Forssk.) Brenan | kenteftafa | shrub |
| Rhamnaceae | *Sageretia thea* (Osbeck) M.C.Johnston | kinchilchile | shrub |
| Ebenaceae | *Euclea racemosa* subsp. *schimperi* (A.DC.) Dandly | kuluo | shrub |
| Berberidaceae | *Berberis holstii* Engl. | mashlaef | shrub |
| Apiaceae | *Heteromorpha trifoliata* (Wendel.) Eckl. and Zeyh. | motsmots deresa/mometse akeytay | shrub |
| Fabaceae | *Cadia purpurea* (Picc.) Ait. | shilen | shrub |
| Lamiaceae | *Clerodendron myricoides* (Hochst.) Vatke | shoha | shrub |
| Sapindaceae | *Dodonaoea angustifolia* L.f. | tahsas | shrub |
| Flacourtiaceae | *Dovyalis verrucosa* (Hochst.) Warb. | tiemtanay | shrub |
| Rubiaceae | *Psydrax schimperiana* (A.Rich.) Bridson | tsehag | shrub |
| Rubiaceae | *Psydrax schimperiana* subsp. *schimperiana* | tselimom | shrub |
| Celastraceae | *Maytenus undata* (Thunb.) Blakelock | atat | shrub |
| Tiliaceae | *Grewia ferruginea* Hochst.exA.Rich. | meleglega | Shrub |
|  |  |  |  |
| **Trees** |  |  |  |
| Sterculiaceae | *Dombeya torrida* (J.F.Gmel.) P. Bamps | bwak | tree |
| Pittosporaceae | *Pittosporum viridiflorum* Sims | myloho | tree |
| Oliniaceae | *Olinia rochetiana* A.Juss. | alalie | tree |
| Anacardiaceae | R. natalensis Krauss | atam | tree |
| Oleaceae | Olea europaea subsp cuspidate (Wall. ex DC.) Cifferri | awlie | tree |
| Fabaceae | Acacia abyssinica Hochst.ex Benth. | cha'a | tree |
| Santalaceae | Osyris quadripartite Decn. | karets | tree |
| Rhizophoracea | Cassipourea malosana (Baker) Alston | keyhom | tree |
| Meliaceae | Ekebergia capensis Sparrm. | kot | tree |
| Ulmaceae | Celtis africana Burm.f. | metekoma | tree |
| Loganiaceae | Nuxia congesta R.Br.ex.Fresen | tekuare | tree |
| Cupressaceae | Juniperus procera Hochst.ex.Endl. | tsihdi adi | tree |
| Cupressaceae | Cupressus lusitanica Miller | tsihdi ferenj | tree |
| Podocarpaceae | Podocarpus (Afrocarpus) falcatus (Thun) Mirb. | zigba | tree |
| Melianthaceae | Bersama abyssinica Fresen. | Mirkuz-zibe | Tree |
| Flacourtiaceae | Dovyalis abyssinica (A.Rich.) Warb. | mongolhats | tree |
| Rutaceae | Teclea simplicifolia (Engl.) Verdoorn | salih | tree |
| Anacardiaceae | Rhus glutinosa A.Rich. | tetalo | tree |
|  |  |  |  |
| **Climber** |  |  |  |
| Vitaceae | Rhoicissus tridentata (L.f.) Wild and Drummond | Keyh-hareg | Woody climber |
